# Correlation Between the Structure and Compressive Property of PMMA Microcellular Foams Fabricated by Supercritical CO_2_ Foaming Method

**DOI:** 10.3390/polym12020315

**Published:** 2020-02-03

**Authors:** Ruizhi Zhang, Ju Chen, Yuxuan Zhu, Jian Zhang, Guoqiang Luo, Peng Cao, Qiang Shen, Lianmeng Zhang

**Affiliations:** 1 State Key Lab of Advanced Technology for Materials Synthesis and Processing, Wuhan University of Technology, Wuhan 430070, China; zhangrz1991@gmail.com (R.Z.); chenju1207@163.com (J.C.); zhuyx_whut@163.com (Y.Z.); zhangjian178@whut.edu.cn (J.Z.); sqqf@263.net (Q.S.); lmzhang@whut.edu.cn (L.Z.); 2 College of Architecture and Civil Engineering, Beijing University of Technology, Beijing 100124, China; caopeng518888@126.com

**Keywords:** cell structure, compressive property, microcellular foams, finite element analysis

## Abstract

In this study, we fabricated poly (methyl methacrylate) (PMMA) microcellular foams featuring tunable cellular structures and porosity, through adjusting the supercritical CO_2_ foaming conditions. Experimental testing and finite element model (FEM) simulations were conducted to systematically elucidate the influence of the foaming parameters and structure on compressive properties of the foam. The correlation between the cellular structure and mechanical properties was acquired by separating the effects of the cell size and foam porosity. It was found that cell size reduction contributes to improved mechanical properties, which can be attributed to the dispersion of stress and decreasing stress concentration.

## 1. Introduction

Foam materials or cellular structure materials are ubiquitous in nature, such as in plants, porcupine quills, bird beaks and feathers; these materials are beneficial for reducing the weight of a structure, and are resistant to flexural and torsional tractions [1,2]. Motivated by their distinctive advantages, lightweight cellular materials have received considerable attention, since they inspire the development of structuring technologies [3,4].

In the aerospace, automotive, marine, rail and wind energy industries, polymeric foam is a promising material as a substitute for conventional metallic components because of its lightweight, good impact energy absorption, thermal insulation and sound absorbance properties [5,6,7].

With polymeric foams finding a variety of applications in numerous industries, it is desirable to correlate their microstructural features with engineering application criteria to optimize manufacturing processes and material selection.

Because of the complex structure, developing an understanding of the correspondence between characteristics of the structure and the mechanical response of foam materials has been proven difficult [8]. The well-known Gibson and Ashby model [9] employs a unit cell with an ideal structural geometry to reasonably predict the mechanical properties (e.g., strength and stiffness) as a function of relative density or porosity of “parent” material.

(1) $$P_{f}=C\cdot P_{s}\cdot {\left(\frac{\rho_{f}}{\rho_{s}}\right)}^{n}$$

According to the equation, the property of the foam material ($P_{f}$) is equal to the property of the “parent” solid material ($P_{s}$) multiplied by the relative density (ρfρs) to the n^th^ power. C and n are two parameters related to the foam architecture, which can be determined experimentally. 

It can be concluded that the physical properties of the foam materials mainly depend on three parameters [10,11]: the intrinsic properties of the “parent” material, the relative density or porosity and the cellular structure. The cellular characters (open or closed), the thickness of struts and cellular walls, and the size, shape and geometric distribution of the cells, determine the cellular structure, i.e., the intrinsic characteristic of the polymer that constitutes the solid framework [12].

The microstructural architecture foam material, e.g., microcellular foam [13], has closed-cell gas bubbles with a cellular diameter of the 10 μm range, and a cell density higher than 10^9^ cells/cm^3^ [10]. It was reported that microcellular foam maintains the mechanical properties of the polymer matrix while increasing the impact toughness [5], if the cell size of the foam is smaller than the critical flaw size of the polymer. Microcellular foams have also demonstrated prior properties [14], such as toughness and fatigue life, when compared with conventional foams [15].

As for other mechanical properties, such as the compressive, tensile and bending properties, researcher opinions differ regarding the advantages of cell size reduction. For example, Wang [16] found that when the foam density or void fraction was the same, the compressive strength and the modulus of the poly (methyl methacrylate) (PMMA) foam increased nearly linearly and exponentially, respectively. Similar results were reported in polyvinylchloride (PVC) [17] and rubber particle-reinforced PMMA foams [18].

However, Brezny and Green [19] found that cell size reduction affects the strut strength. These authors investigated the structure and mechanical properties of brittle carbon foams. The results show that the compressive elastic modulus and fracture toughness of carbon foams were insensitive to the variation in the cell size at the same foam porosity, while the crushing and bending strength change inversely with the changing cell size. Weller [20] investigated the effect of the cell size on the tensile behavior of high relative density or low porosity microcellular polycarbonate foams, and concluded that the tensile modulus, tensile strength, elongation to break, and the toughness, were not affected by the average cell size within the range from 4 to 40 μm. Doroudiani [21] built the tensile property–structure relationship of expanded microcellular polystyrenes (EPS) based upon statistical experiment design, and concluded that there was no significant influence of cell size on the tensile properties of EPS foams. Alvarez et al. [12] explored the relationships between the structure and mechanical properties of high-density foams via a finite element model (FEM). The results show that foams with average cell sizes of 10 mm and 100 mm show a similar modulus at the same density; thus, these researchers concluded that the cell size has a negligible influence on the elastic modulus of the foams.

In summary, the correlation between various mechanical properties and the foam structure has not been explicitly investigated.

During experimental testing, separating the effects of the microstructure architecture from the foam density remains exceedingly difficult. It is exceedingly challenging to isolate the two coupled parameters experimentally, as typical foaming methods allow for only limited control of the porosity of the “parent” material. Nevertheless, during the curing process, the cellular architecture always changes with the density [22].

The supercritical fluid foaming method is regarded as the most convenient way to synthesize microcellular foam. Moreover, this method may be the most likely way to regulate the foam density and the cellular architecture, as it has fewer procedures, and the foaming agents have a small effect on the “parent” material compared with traditional mechanical and chemical foaming methods.

Studies of the effect of the cell size or foam density on the mechanical properties are still scarce. Wang [16] studied the dependence of mechanical properties on the cellular structure of the PMMA foams by independently analyzing the effects of the cell size and the void fraction. However, the discussion was limited to a void fraction of 0.95, with no discussion of other densities or fractions. Moreover, the underlying reasons for the structure effect have rarely been discussed [18,23,24,25].

In this article, a systematic study takes into account the foam porosity and cellular structure to determine the correlation with the foam properties. A series of PMMA microcellular foams with foam porosity ranging from 36.5% to 85.8% and cell sizes ranging from 2.42 to 52.4 μm were fabricated by the supercritical CO_2_ foaming method. 

By carefully choosing the sample, the parameters of the foam porosity and cell size were separated, and the compressive properties were tested. Moreover, a numerical analysis based on the FEM was adopted to further demonstrate the experimental results and failure mechanism.

## 2. Experimental

### 2.1. Chemicals and Foaming Production

Poly (methyl methacrylate) (PMMA) (MF001) was purchased from Mitsubishi Chemical Polymer Nantong Co., Ltd., Nantong, China. The PMMA had a weight-average molar mass (M_w_) of 110,000 g/mol and a density (ρ) of 1.19 g/cm^3^. The glass transition temperature (T_g_) of the PMMA was 105.5 °C, which was determined by differential scanning calorimetry (DSC2500, TA Instruments). Commercial purity grade CO_2_ with a purity of 99% was supplied by Xiangyun Co., Ltd., Wuhan, China, serving as a blowing agent for the supercritical foaming method.

Supercritical foaming experiments were processed in a self-designed supercritical fluid foaming system (Beijing Xingda Co., Ltd., Beijing, China). The CO_2_ pressure and system temperature were controlled by two high-precision syringe pumps (ISCO Model 260D) and a proportional–integral–derivative (PID)-based magnetic stirring mixer heating system, respectively.

Before the foaming method, the PMMA samples were pretreated in an air-dry oven at 80 °C for 12 h and then carved at the desired dimensions and thickness by a carving machine. During the foaming process, the dense PMMA samples were placed into the high-pressure vessel filled with CO_2_ under designed pressure and temperature conditions. 

A collection of experiments, including four different foaming temperatures (50, 80, 110, 130 °C) and six different foaming pressures (8, 13, 18, 23, 28, 32 MPa) were performed using a three-stage process. In the first stage, the samples were saturated under fixed gas pressure and die temperature conditions for at least 8 h. The next stage was a desorption process, in which the pressure decreased to room pressure in less than 10 s, and the gas in the polymer began to nucleate and grow. The last stage was cooling, in which the plasticized samples were cooled to a lower temperature than the effective glass transition temperature. During this stage, the samples solidified, and the bubbles were fixed.

To guarantee that the foamed samples remained flat for the following mechanical experiments, a perforated aluminum sheet mold was applied to constrain the solid PMMA samples during the foaming procedures. The sheets were specially designed, and the spring was loaded to allow for the free expansion of the samples without warpage [26].

### 2.2. Characterization and Testing

#### 2.2.1. Density Determining

Before testing, the samples were placed at room temperature for at least one week for the elimination the residual CO_2_ in the bubbles.

The apparent densities (ρ) of the foamed and solid samples were measured using a helium pycnometer (TD2200, Beijing Biaode Electron Technology Co., Ltd., Beijing, China) and density determination kit for the analytical balance (Mettler Toledo ML204). At least three measurements were conducted for each sample.

The relative density ($\rho_{r}$) and porosity (∅) of the foamed samples were calculated using Equations (2) and (3), respectively:

(2) $$\rho_{r}=\frac{\rho_{f}}{\rho_{s}}$$

(3) $$\emptyset =1-\rho_{r}$$

#### 2.2.2. Microstructural Observation

Before observation, the samples were freeze–fractured in liquid nitrogen and cut using a sharp razor blade to obtain cross sections of the samples. The fracture surfaces were sputter coated with gold. A field emission scanning electron microscopy (FESEM, Quanta–250) was utilized to observe the morphology of the samples. Cell density and cell size analyses were conducted using the ImageJ program (National Institute of Health), and more than 100 cells were tested. The cell density was calculated using Equation (4):

(4) $$N={\left(\frac{nM^{2}}{A}\right)}^{3/2}$$

where N is the cell density (cells/cm^3^), A is the area of the SEM image, M is the magnification factor, and n is the number of cells counted from the SEM images.

#### 2.2.3. Compressive Property Testing

Cubic specimens with a nominal length of 5 mm were prepared for testing according to the American Society for Testing and Materials (ASTM) standard D695 for plastics at room temperature. Compressive properties were tested using the Shanghai Qingji electron universal mechanical testing machine with an Instron model QJ210A. At least three specimens are tested for each kind of sample at a velocity of 0.5 mm·min^−1^ at room temperature.

#### 2.2.4. FEM Simulation

The evaluation of the elastic response of the modeled structures was performed using ABAQUS finite element analysis. Three-dimensional representative volume element (RVE) models based on a representative structural unit comprised of a set of randomly distributed cells were applied. RVE models more exactly reproduce real foams, since they consider mesoscopic features that directly affect the final mechanical behavior [27,28]. It was assumed that the average mechanical properties of the RVE models were equal to the average properties of the particular foams. The RVE model of the low-density foam was investigated using the Voronoi tessellation technique, while the model of high-density foam was analyzed using a unit cell technique. The material parameters of the damaged plasticity model in ABAQUS were calibrated based on the test results. A linear elastic constitutive model was used for the PMMA solid, with a Young’s modulus of 1100 MPa and a Poisson ratio of 0.391.

## 3. Results and Discussion

### 3.1. Foam Porosity

The experimental design to investigate the relationship between the processing conditions and the foam density are summarized in Table 1.

**Figure 1:**
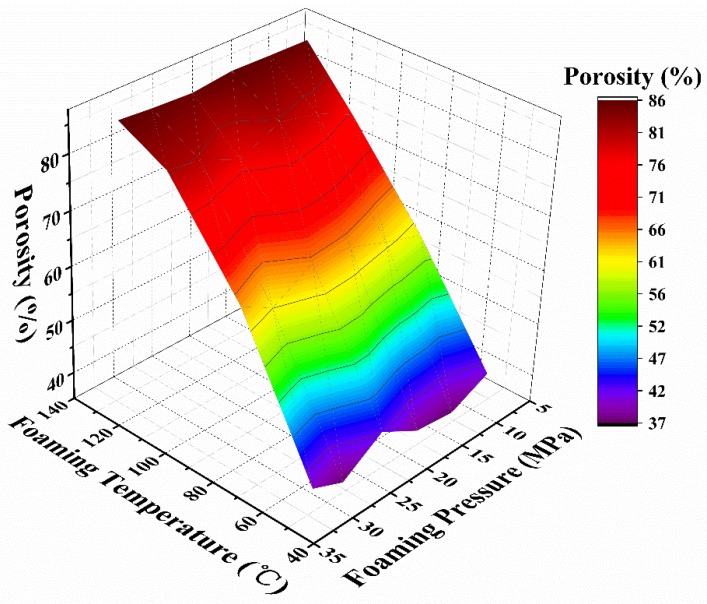
Foam porosity as a function of the foaming parameters.

Table 1 and Figure 1 summarize the foam density and porosity as a function of the foaming parameters. It was demonstrated that in the designed foaming parameters, the foam density can be tailored in the range of 0.17–0.75 g·cm^−3^ by adjusting the foaming conditions. Accordingly, the porosity varies from 36.5% to 85.8%. The density or porosity seems to be independent of the foaming pressure, but is largely affected by the foaming temperature. In the high temperature region, the porosity becomes stable, regardless of the foaming pressure. The experimental data of the foam porosity (∅) and the foaming temperature (T) in Figure 1 can be fitted by a linear equation, as shown in Equation (5).

(5) ∅=8.91+0.62T

### 3.2. Foam Microstructure

**Figure 2:**
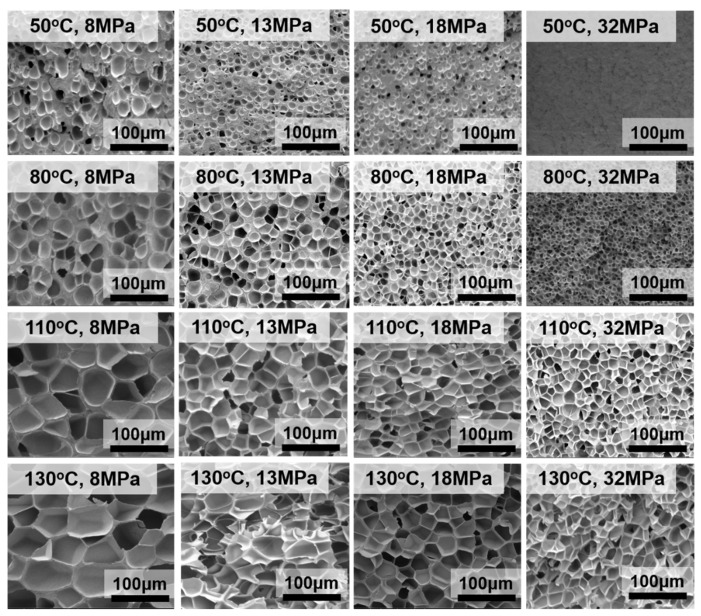
SEM images of typical foamed samples synthesized under various foaming conditions.

The microstructure of representative foamed samples is shown in Figure 2. To render a clear comparison, all micrographs were placed at the same magnification. Notably, all the foams show a typical closed-cell structure, as previously reported [29]. The shape of the cells changes from a sphere to a polygon as the temperature increases. The foaming temperature is the main factor contributing to the changes in the cell size. At a certain foaming temperature, the foaming pressure has a greater effect on the cell size in the low temperature region than that in the high temperature region.

**Figure 3:**
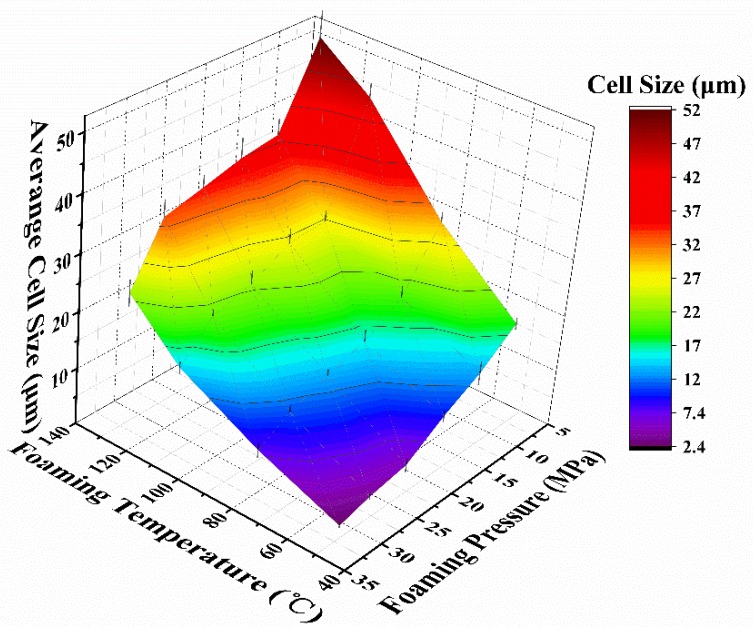
Cell size of the foamed samples as a function of the foaming parameters.

**Figure 4:**
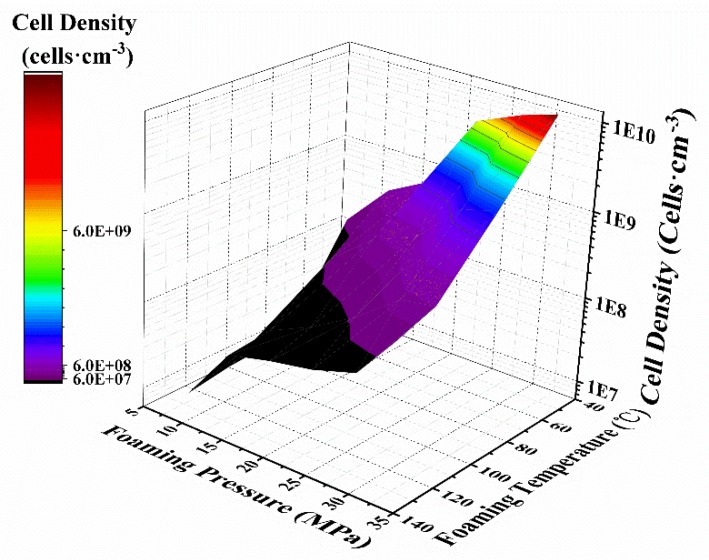
Cell density of the foamed samples as a function of the foaming parameters.

To quantitatively analyze the cell structure, the cell size and cell density data are presented in Figure 3 and Figure 4, respectively.

As shown in Figure 3 and Figure 4, both the foaming temperature and pressure affect the cell size and cell density, which ranges from 2.42 to 52.4 µm and 1.22 × 10^10^ to 8.39 × 10^6^ cells·cm^−3^, respectively. These results also demonstrate that the cell structures are greatly improved by decreasing the foaming temperature, and that the relationship is nearly linear. In contrast, as the foaming pressure increases, the cell size decreases, and the cell density increases, and the relationship between cell size, cell density and foaming pressure is nonlinear. 

The foaming temperature affected many parameters of both the PMMA matrix and foaming agents, such as the CO_2_ content, diffusion rate and rheological property [16,24,30]. At a lower foaming temperature, the PMMA polymer melt presents a larger viscosity and a higher stiffness, so it is difficult for the cells to surmount the resistance. As the foaming temperature increases, the stiffness and viscosity of the polymer gradually decrease, which is beneficial for cell growth, thus leading to a high porosity (Figure 1).

The influence of the foaming pressure on the cellular microstructure can be attributed to the retrograde vitrification phenomenon in the PMMA-CO_2_ system; that is, as the weight of absorbed CO_2_ increases, the Tg decreases, and the CO_2_ has a lower barrier to growth [31,32]. Moreover, according to classical nucleation theory, a higher quench pressure or foaming pressure leads to a high nucleation rate, resulting in a higher cell density [33,34].

According to the classical nucleation theory, the foaming pressure has a remarkable effect on the nucleation barrier. That is, the heterogeneous nucleation barrier (ΔG*) more significantly increased with decreasing saturation pressure (ΔP). In other words, when the foaming pressure is increased, the barrier value drastically reduced. Therefore, the cell density increases accompanied by a drop in the cell size [35].

**Figure 5:**
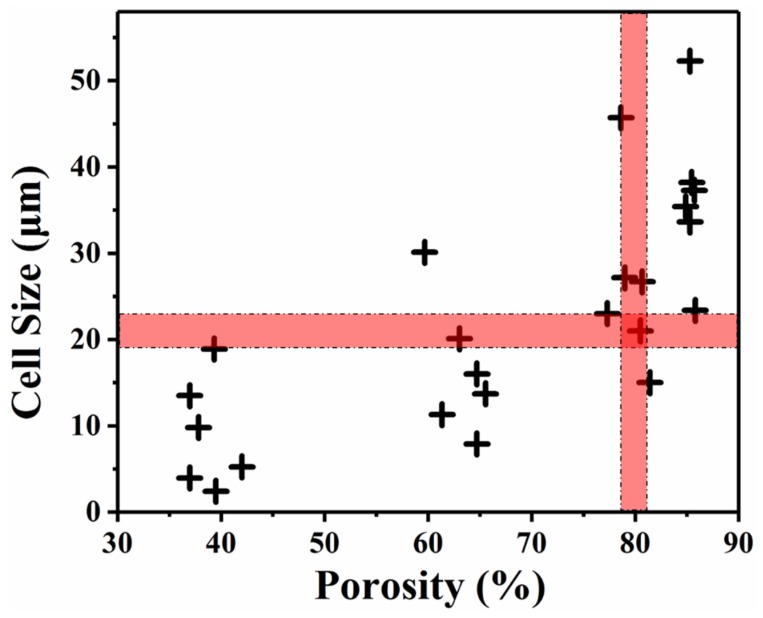
Cell size of foamed sample with a relationship of its porosity.

By combining the cellular structure data and porosity data, the relationship between the cell size and the porosity is plotted in Figure 5. The result shows a wide range of data for both the cell size and porosity. From Figure 5, foams of the same porosity with different cell sizes, and foams of the same cell size with different porosities, can be easily identified. The cell size and porosity can be separated for further individual study.

### 3.3. Mechanical Properties

**Figure 6:**
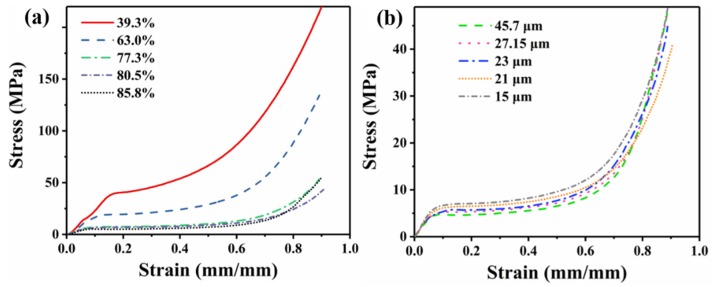
Stress–strain curves of the foamed sample as a function of its porosity and cell size: (**a**) cell size of nearly 20 μm and (**b**) porosity of nearly 82%.

**Figure 7:**
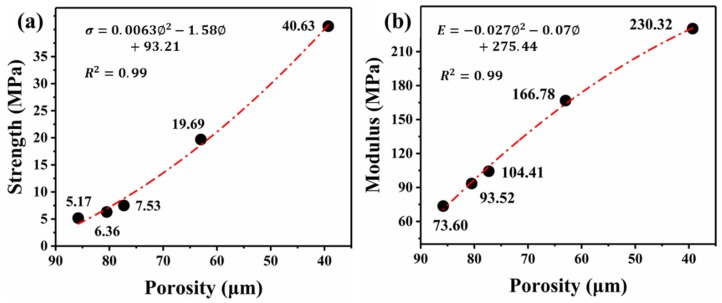
Compressive strength (**a**) and modulus (**b**) of the foam as a function of its porosity (cell size of nearly 20 μm).

**Figure 8:**
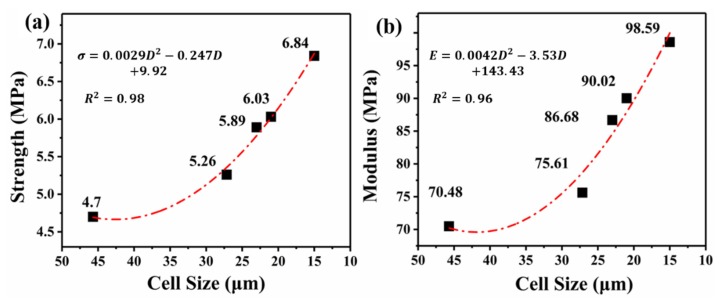
Compressive strength (**a**) and modulus (**b**) of the foam as a function of its cell size (porosity of nearly 82%).

Figure 6 displays the stress–strain curves of a foamed sample as a function of its porosity and cell size, and the relative strength and modulus data are shown in Figure 7 and Figure 8, respectively. It can be seen from Figure 6 that all of the foams display three typical stress–strain regions of the cellular materials. Compared with low-porosity foams, high-porosity foams have a lower plateau strength and a longer plateau region (Figure 6a)). There was a slight difference between foams with different cell sizes (Figure 6b)). 

Expectedly, both the strength and modulus increase with decreasing porosity (Figure 7). This phenomenon can be attributed to the gradually gained solid material in both the cell struts and cell walls with a reduction in porosity [25].

The relations between the porosity (∅) and the strength (σ) and modulus (E) show quadratic relations.

(6) $$\sigma =0.0063\emptyset^{2}-1.58\emptyset +93.21$$

(7) $$E=-0.027\emptyset^{2}-0.07\emptyset +275.44$$

For foams with nearly 82% porosity, the stress–strain curves exhibit the same trend (Figure 8). With a cell size reduction from 45.7 to 15 μm, there was a slight increase in the strength from 4.7 to 6.84 MPa, and modulus from 70.48 to 98.59 MPa. Additionally, there was an increase of 2.14 MPa in the compressive strength, and the compressive modulus is increased by more than 40%. The correlation between the cell size and the compressive strength and modulus can be fitted by Equations (8) and (9).

(8) $$\sigma =0.0029D^{2}-0.247D+9.92$$

(9) $$E=0.0042D^{2}-3.53D+143.43$$

Interestingly, from Figure 8 and Equations (8) and (9), when the cell size is greater than 30 μm, with an increasing cell size, the decrease rate of the strength and modulus is much slower. In other words, the strength and modulus gain a greater enhancement in smaller cell size foams. A similar quadratic curve was reported in previous literature [36]. When cell diameters are greater than 30 μm, the shape of the cells is a polygon, and the wall thickness is very thin, and the struts fraction of the polymer matrix is low. Then the cell wall mainly withstands the pressure. As the cell diameter gradually increases, the wall thickness decreases at a slower rate, which leads to the tiny reduction of mechanical strength.

### 3.4. Numerical Simulation on Mechanical Properties

To theoretically analyze the effect of the cell size on the mechanical properties of PMMA microcellular foams, a model for predicting the mechanical properties and describing the evaluation of cell structure was established based on the multiscale theory. From a mesoscopic perspective, the PMMA microcellular foams are equivalent to dense PMMA and cell composites, of which the PMMA is the matrix and cells are the inclusions. The mechanical properties of each equivalent medium with different porosities and cell sizes can be predicted.

**Figure 9:**
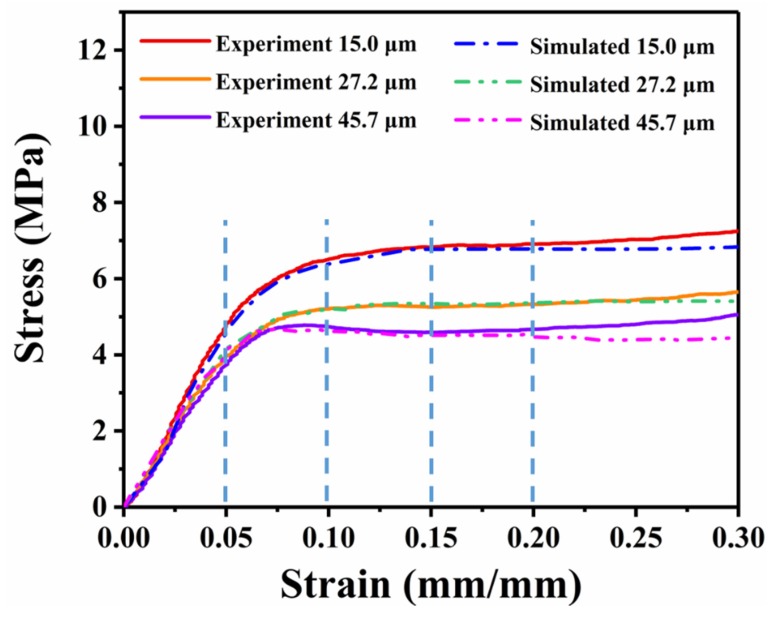
Normalized stress–strain curves of the experimental and simulated results of foams with the same porosity of nearly 82%.

Figure 9 compares the normalized experimental and simulated stress–strain responses in the elastic and plateau regions. Three different experimental results of Figure 6b were chosen. The original stress–strain curves obtained by FEMs are shown with discontinuous thin lines. The curves obtained by the nonlinear homogenization approach are displayed as a dashed dotted line. The good agreement between the experimental data and numerical simulation data indicates that the proposed multiscale model is suitable for simulating the compressive strength of the foams. 

**Figure 10:**
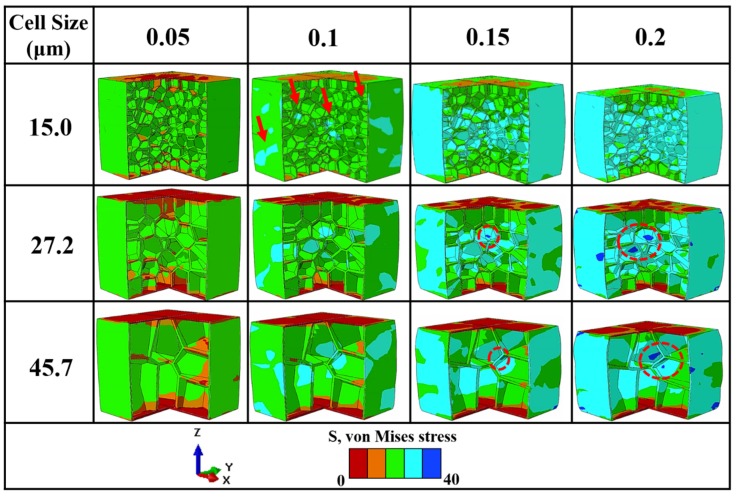
Three-dimensional (3D) stress contour maps for foams of the same porosity of 82% under quasi-static loading.

Figure 10 demonstrates the xz- and yz-cross sections of the 3D stress contour strain maps, which were extracted from Figure 9 at four different strains. At the elastic stage (0.05 strain), all of the foam samples deform via an elastic deformation of the foam skeleton, yielding a uniform stress field with a low amplitude, where the contour maps are in the same color. When the strain increases to 0.1, it is apparent that the stress concentration occurs in the cell walls; however, the foam with a cell size of 45.7 μm shows a larger localization area and higher stress level.

In contrast, the 15 μm foam structure triggers a discrete stress concentration zone in both the center and outer areas, as indicated by the arrows. By applying an additional load, the deformation banding connects in the center region, and a significant stress concentration nucleation is observed. The cell walls nearby begin to buckle.

To summarize, FEM computation was capable of qualitatively analyzing the deformation behavior of foams with different cell sizes. The main difference in the failure mechanism among the three kinds of foams is that the stress concentration is easily generated and coalesced in the walls or struts of large cell sizes, where failure occurs. For foams with smaller cell sizes, the interlaced cellular structure strengthens each other and homogenizes the stress concentration.

## 4. Conclusions

In this work, several PMMA microcellular foams with various porosities in the range of 36.5% to 85.8% and cellar structures ranging from 2.42 to 52.4 μm were fabricated by the ScCO_2_ foaming method. Samples with the same porosity of 82% and the same cell size of 20 μm were chosen for investigating mechanical properties. The correlation between the compressive properties and cellular structure was obtained. Either decreasing the porosity or reducing the cell size can increase the compressive strength and modulus. FEM modeling connected the microscale response with bulk performance, and demonstrated that a smaller cell size or higher cell density will facilitate stress transfer and decrease the stress concentration, contributing to higher compressive strength. 

Many possibilities exist for future work. More microscale factors such as cell regularity, struts fractions and cell size grading can be included to guide structure design for high performance foam materials.

## Figures and Tables

**Table 1:** Foam density, porosity, cell size and cell density results of samples at various foaming parameters.

Foaming Temperature (°C)	Saturation Pressure (MPa)	ρ (kg/m^3^)	Porosity (%)	Cell Size (μm)	Cell Density (cells·cm^−3^)
50	8	0.722 ± 0.039	39.3 ± 3.26	18.6 ± 3.01	(1.41 ± 0.34) E08
	13	0.751 ± 0.027	36.9 ± 2.31	13.5 ± 2.13	(4.55 ± 0.26) E08
	18	0.740 ± 0.025	37.8 ± 2.12	9.80 ± 3.05	(8.30 ± 0.97) E08
	23	0.690 ± 0.038	42.0 ± 3.21	5.24 ± 2.28	(5.74 ± 0.86) E09
	28	0.755 ± 0.013	36.5 ± 1.13	3.96 ± 0.53	(9.29 ± 0.53) E09
	32	0.719 ± 0.027	39.5 ± 2.31	2.42 ± 0.42	(1.22 ± 0.83) E10
80	8	0.481 ± 0.052	59.6 ± 4.35	30.1 ± 2.74	(1.13 ± 0.33) E07
	13	0.440 ± 0.064	63.0 ± 5.42	20.1 ± 2.08	(1.36 ± 0.15) E08
	18	0.421 ± 0.052	64.7 ± 4.33	16.0 ± 2.12	(3.40 ± 0.29) E08
	23	0.409 ± 0.035	65.6 ± 2.98	13.7 ± 1.06	(5.23 ± 0.38) E08
	28	0.460 ± 0.049	61.3 ± 4.15	11.3 ± 2.25	(1.56 ± 0.64) E09
	32	0.422 ± 0.012	64.7 ± 1.05	7.90 ± 1.32	(1.99 ± 0.13) E09
110	8	0.260 ± 0.043	78.2 ± 3.62	45.7 ± 8.41	(1.01 ± 0.25) E07
	13	0.254 ± 0.059	78.7 ± 4.96	27.2 ± 1.89	(4.15 ± 0.42) E07
	18	0.232 ± 0.014	80.5 ± 1.21	26.7 ± 2.46	(6.93 ± 0.68) E07
	23	0.270 ± 0.063	77.3 ± 5.32	23.0 ± 2.35	(1.34 ± 0.17) E08
	28	0.232 ± 0.040	80.5 ± 3.34	21.1 ± 1.20	(3.18 ± 0.26) E08
	32	0.221 ± 0.051	81.4 ± 4.32	15.2 ± 1.15	(3.73 ± 0.35) E08
130	8	0.175 ± 0.024	85.3 ± 2.02	52.4 ± 8.52	(8.39 ± 0.33) E06
	13	0.173 ± 0.014	85.5 ± 1.17	38.3 ± 2.84	(3.68 ± 0.28) E07
	18	0.170 ± 0.034	85.7 ± 2.98	37.3 ± 7.46	(4.82 ± 0.17) E07
	23	0.183 ± 0.043	84.6 ± 3.58	35.4 ± 7.12	(5.92 ± 0.55) E07
	28	0.175 ± 0.024	85.3 ± 2.05	33.6 ± 6.02	(8.39 ± 0.48) E07
	32	0.169 ± 0.033	85.8 ± 2.81	23.4 ± 5.34	(2.56 ± 0.97) E08
